# Measuring spirituality and religiosity in clinical research: a systematic review of instruments available in the Portuguese language

**DOI:** 10.1590/S1516-31802013000100022

**Published:** 2013-04-01

**Authors:** Giancarlo Lucchetti, Alessandra Lamas Granero Lucchetti, Homero Vallada

**Affiliations:** I MD, PhD. Specialist in Geriatrics. Adjunct Professor, Department of Medicine, Universidade Federal de Juiz de Fora (UFJF), Juiz de Fora, Minas Gerais, Research Collaborator of João Evangelista Hospital and São Paulo Medical Spiritist Association, São Paulo, Brazil.; II MD. Specialist in Geriatrics. Member of the Research Department of São Paulo Medical Spiritist Association, João Evangelista Hospital and MBA Student at Fundação Getúlio Vargas, São Paulo, Brazil.; III MD, PhD. Specialist in Psychiatry. Associate Professor, Department of Psychiatry, Faculdade de Medicina da Universidade de São Paulo (FMUSP), São Paulo, Brazil.

**Keywords:** Religion and medicine, Spirituality, Psychometrics, Review [publication type], Medical history taking, Religião e medicina, Espiritualidade, Psicometria, Revisão, Anamnese

## Abstract

**CONTEXT AND OBJECTIVES:**

Despite numerous spirituality and/or religiosity (S/R) measurement tools for use in research worldwide, there is little information on S/R instruments in the Portuguese language. The aim of the present study was to map out the S/R scales available for research in the Portuguese language.

**DESIGN AND SETTING:**

Systematic review of studies found in databases.

**METHODS:**

A systematic review was conducted in three phases. Phases 1 and 2: articles in Portuguese, Spanish and English, published up to November 2011, dealing with the Portuguese translation and/or validation of S/R measurement tools for clinical research, were selected from six databases. Phase 3: the instruments were grouped according to authorship, cross-cultural adaptation, internal consistency, concurrent and discriminative validity and test-retest procedures.

**RESULTS:**

Twenty instruments were found. Forty-five percent of these evaluated religiosity, 40% spirituality, 10% religious/spiritual coping and 5% S/R. Among these, 90% had been produced in (n = 3) or translated to (n = 15) Brazilian Portuguese and two (10%) solely to European Portuguese. Nevertheless, the majority of the instruments had not undergone in-depth psychometric analysis. Only 40% of the instruments presented concurrent validity, 45% discriminative validity and 15% a test-retest procedure. The characteristics of each instrument were analyzed separately, yielding advantages, disadvantages and psychometric properties.

**CONCLUSION:**

Currently, 20 instruments for measuring S/R are available in the Portuguese language. Most have been translated (n = 15) or developed (n = 3) in Brazil and present good internal consistency. Nevertheless, few instruments have been assessed regarding all their psychometric qualities.

## RESUMO

**CONTEXTO E OBJETIVOS:**

Apesar do grande número de escalas sobre espiritualidade e religiosidade (E/R) para uso em pesquisas internacionais, ainda existem poucas informações sobre essas escalas na língua portuguesa. Objetiva-se mapear as escalas disponíveis para pesquisa clínica em língua portuguesa.

**TIPO DE ESTUDO E LOCAL:**

Revisão sistemática de estudos encontrados em bases de dados.

**MÉTODOS:**

Conduziu-se uma revisão sistemática em três fases. Fases 1 e 2: artigos em português, espanhol e inglês, publicados até novembro de 2011, com a tradução ou validação para o português de instrumentos para aferir E/R na pesquisa clínica, foram selecionados de seis bancos de dados. Fase 3: os instrumentos foram agrupados de acordo com autoria, adaptação transcultural, consistência interna, validade concorrente/discriminativa e procedimentos de teste-reteste.

**RESULTADOS:**

Vinte instrumentos foram encontrados. Destes, 45% avaliavam religiosidade, 40% espiritualidade, 10% coping (enfrentamento) religioso/espiritual e 5% espiritualidade e religiosidade. Destes, 90% foram traduzidos (n = 15) ou criados (n = 3) para a língua portuguesa no Brasil e 2 (10%) somente para o português de Portugal. Entretanto, a maioria dos instrumentos não possuía análises psicométricas. Apenas 40% dos instrumentos apresentavam validação concorrente, 45% validação discriminativa e 15% teste-reteste. A característica de cada instrumento foi analisada de forma individualizada com vantagens, desvantagens e propriedades psicométricas.

**CONCLUSÃO:**

No momento, existem 20 instrumentos para aferição de E/R disponíveis para a língua portuguesa. A maioria é traduzida (n = 15) ou desenvolvida (n = 3) no Brasil apresentando boa consistência interna. Entretanto, poucas escalas foram avaliadas quanto a todas as suas qualidades psicométricas.

## INTRODUCTION

There is growing interest in the field of spirituality/religiosity (S/R) and its relationship to health. Studies have shown that individuals with higher levels of S/R have lower prevalence of depression and anxiety, better quality of life, lower prevalence of cardiological problems and lower mortality.^1^^,^^2^^,^^3^

Nevertheless, empirical S/R evidence has been subject to criticism for several reasons, such as failure to control for confounding variables, failure to control for multiple comparisons, conflicting findings and an excessive number of instruments and approaches for measuring S/R.^4^ In fact, measuring spirituality in clinical practice and research has posed a particular challenge because of the complexity of the elements and definitions involved. Since there is no widely accepted approach for measuring spirituality,^5^ a wide range of S/R research instruments has emerged.

Recently, two reviews were conducted evaluating the religiosity^6^ and spirituality^7^ instruments/tools available worldwide. These reviews found that the tools measured an array of different dimensions, including organizational religiosity, non-organizational religiousness, religious/spiritual coping, intrinsic religiousness, beliefs and values, religious affiliation, religious struggle, spiritual wellbeing, general spirituality and spiritual needs, among others.

Despite the large number of different measurement instruments in use worldwide, there is little information on S/R instruments in the Portuguese language. In order to consolidate this field of research, it is important to have effective and validated instruments available for use. Therefore, an analysis on the instruments available in the Portuguese language and their psychometric properties may foster discussions on this issue and encourage further studies.

## OBJECTIVES

The aim of the present study was to map out the S/R measurement scales available in the Portuguese language.

## METHODS

A systematic review was conducted to gather information about the scales/tools designed to measure S/R that had previously been translated into Portuguese. This paper uses the term systematic review to denote the entire process of retrieval, selection, appraisal, summarizing and reporting of evidence.

### Search strategies

The data abstraction entailed three phases, as described below.

Phase 1 (primary literature search): two researchers (GL, ALGL) independently screened the list of references (full articles were retrieved for further analysis whenever necessary) to exclude studies that did not address the issue at hand. Any disagreements between the reviewers were discussed with a third reviewer (HV) and resolved by reaching a consensus.

Articles in Portuguese, Spanish and/or English dealing with the Portuguese translation and/or validation of S/R tools for scientific research, published up to November 2011, were selected.

Articles dealing with translation or validation of S/R scales in languages other than Portuguese, as well as review articles only citing the scales, were excluded. All articles not fulfilling the inclusion criteria and which met the exclusion criteria were omitted from the final analysis.

The following databases were evaluated: PubMed (http://www.pubmed.gov.br); Excerpta Medica (EMBASE) (www.embase.com); Cochrane Library (http://www.thecochranelibrary.com/); Latin American and Caribbean Health Sciences Literature (Literatura Latino-Americana e do Caribe em Ciências da Saúde, Lilacs) (http://www.bireme.br); and Scientific Electronic Library Online (SciELO), which is a database involving Portuguese and Spanish language-speaking countries (http://www.scielo.br).

The keywords used (Table 1) were as follows:

- in English: (a) (Spiritual* AND instruments AND Brazil) OR (Spiritual* AND instruments AND Portug*); (b) (Religio* AND instruments AND Brazil) OR (religio* AND instruments AND Portug*); (c) (Religio* AND scale AND portug*) OR (religio* AND scale AND Brazil); (d) (Spiritual* AND scale AND portug*) OR (spiritual* AND scale AND Brazil); (e) (Religio* AND index AND portug*) OR (religio* AND index AND Brazil); (f) (validation AND spiritual* AND portug*) OR (validation AND spiritual* AND Brazil); (g) (Validation AND religio* AND Portug*) OR (validation AND religio* AND Brazil).
- in Portuguese: (a) Instrumentos AND espiritualidade; (b) Instrumentos AND religiosidade; (c) Escala AND religiosidade; (d) Escala AND espiritualidade; (e) Índice AND religião AND Brasil; (f) Validação AND espiritualidade; (g) Validação AND religiosidade.

**Table 1.** Data abstraction (phase 1 - primary literature search)

Keywords	PubMed		EMBASE*		Cochrane Library*		SciELO*		Lilacs*	
	Total	Included	Total	Included	Total	Included	Total	Included	Total	Included
(Spiritual* AND instruments AND Brazil) OR (Spiritual* AND instruments AND Portug*)	4	2	7	0	0	0	9	0	2	0
Instrumentos AND espiritualidade	-	-	-	-	-	-	8	1	12	0
(Religio* AND instruments AND Brazil) OR (religio* AND instruments AND Portug*)	5	0	16	0	0	0	9	0	9	0
Instrumentos AND religiosidade	-	-	-	-	-	-	7	0	12	1
(Religio* AND scale AND portug*) OR (religio* AND scale AND Brazil)	10	1	136	1	0	0	23	0	5	1
Escala AND religiosidade	-	-	-	-	-	-	13	0	14	2
(Spiritual* AND scale AND portug*) OR (spiritual* AND scale AND Brazil)	7	0	18	0	0	0	13	2	9	0
Escala AND espiritualidade	-	-	-	-	-	-	15	1	59	3
(Religio* AND index AND portug*) OR (religio* AND index AND Brazil)	21	1	261	0	0	0	5	0	8	0
Índice AND religião AND Brasil	-	-	-	-	-	-	6	0	10	0
(Validation AND spiritual* AND portug*) OR (validation AND spiritual* AND Brazil)	2	0	5	0	0	0	6	0	52	0
Validação AND espiritualidade	-	-	-	-	-	-	4	0	4	0
(Validation AND religio* AND Portug*) OR (validation AND religio* AND Brazil)	2	0	31	0	0	0	2	0	2	0
Validação religiosidade	-	-	-	-	-	-	1	0	5	0
(Spiritual* AND measur* AND Brazil) OR (Spiritual* AND measur* AND Portug*)	4	0	12	0	0	0	0	0	1	0
Medida AND espiritualidade	-	-	-	-	-	-	5	0	5	0
(Religio* AND measur* AND Brazil) OR (Religio* AND measur* AND Portug*)	21	0	200	0	0	0	0	0	2	0
Medida AND religiosidade	-	-	-	-	-	-	8	0	9	0
Total	76	4	686	1	0	0	134	4	220	7

*Duplicate articles in these databases were not included.

Phase 2 (manual literature search): A manual search of the literature was conducted as an additional phase of the search process, with the aim of identifying studies that were missed in the primary search. Since there seems to be no standard practice with regard to conducting manual literature searches, and in order to increase the search sensitivity, the names of specific scales were used as keywords. These scales were chosen based on those reported by a previous review (conducted by Koenig et al.^6^ to investigate the most common S/R scales used for research) that was not found in Phase 1.

The following databases were then evaluated: PubMed (http://www.pubmed.gov.br); EMBASE (www.embase.com); Cochrane Library (http://www.thecochranelibrary.com/), Lilacs (http://www.bireme.br); SciELO (http://www.scielo.br); and Google Scholar (www.scholar.google.com). Only the first 100 references from Google Scholar were evaluated. Google Scholar was included only in this phase because, according to recent studies, “Google Scholar, as for the Web in general, can help in the retrieval of even the most obscure information but its use is marred by inadequate, less often updated, citation information”^8^.

The keywords used were as follows:

- (The Santa Clara Strength of Religious Faith Questionnaire) AND (Portug* OR Brazil)
- (Systems of Belief Inventory) AND (Portug* OR Brazil)
- (FACIT sp 12) AND (Portug* OR Brazil)
- (Inspirit-R) AND (Portug* OR Brazil)
- (Daily Spiritual Experiences) AND (Portug* OR Brazil)
- Fetzer/NIA Multidimensional Measurement of Religiousness/Spirituality AND (Portug* OR Brazil).

Phase 3 (critical review of instruments): the articles were evaluated taking the following factors into consideration:

- Article characteristics: authors, year of publication and publishing journal.
- Instrument validation process:
  - translation process: consisting of (a) forward translation, i.e. translation of the original language (also called the source language) version of the instrument into another language (often called the target language); and (b) back translation, i.e. translation of the new language version back into the original language;
  - cross-cultural adaptation: if an instrument was previously validated, this does not necessarily mean that it is valid for use in another time period, culture or context. Therefore, it is necessary to adapt instruments used in other cultural settings. As an example, a questionnaire that asks about physical activity and uses cross-country skiing as an example may not be relevant in settings where there is no snow;
  - internal consistency: this is the extent to which tests or procedures assess the same characteristic, skill or quality. It is a measure of the precision among observers or measuring instruments used in a study;
  - concurrent/convergent validity: this is a measure of the degree to which a given test correlates with a previously validated measurement;
  - discriminative/discriminant validity: this examines the extent to which a measurement correlates with measures of attributes that differ from the attribute that this measurement is designed to assess;
  - test-retest procedure: this is the variation in measurement when taken by a single person or instrument on the same item and under the same conditions;
- Setting evaluated: sample characteristics and number of participants.

## RESULTS

### Data abstraction

**Figure 1.**
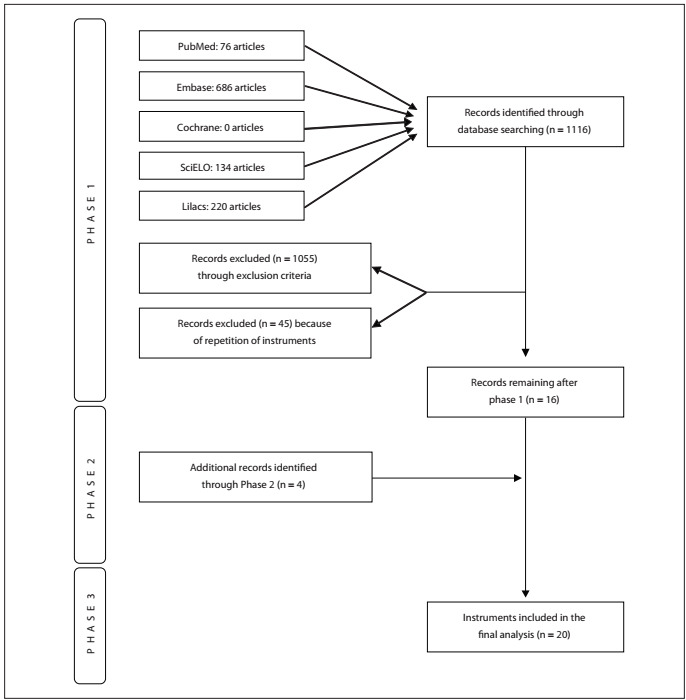
PRISMA (The Preferred Reporting Items for Systematic Reviews and Meta-Analyses) flow diagram.

Phase 1 (primary literature search): use of the keywords led to retrieval of 76 articles from PubMed (4 included), 686 from EMBASE (1 included), none from the Cochrane Library (none included) and 134 from SciELO (4 included) and 220 from Lilacs (7 included), giving a total of 16 instruments in this phase (Table 1 and Figure 1).

Phase 2 (manual literature search): using scale names as keywords, six scales were examined and four of these were included in the study (Table 2).

**Table 2.** Data abstraction (phase 2 - manual literature search)

Scale	Databases
(The Santa Clara Strength of Religious Faith Questionnaire) AND (Portuguese OR Brazil)	Found
(Systems of Belief Inventory) AND (Portuguese OR Brazil)	Not found
(FACIT sp 12) AND (Portuguese OR Brazil)	Found
(Inspirit-R) AND (Portuguese OR Brazil)	Found
(Daily Spiritual Experiences AND (Portuguese OR Brazil)	Found
Fetzer/NIA Multidimensional Measurement of Religiousness/Spirituality AND (Portuguese OR Brazil)	Not found

Phase 3 (critical review of instruments): in this phase, all 20 instruments found were evaluated in terms of authors, year of publication, publishing journal, translation process, cross-cultural adaptation, internal consistency, concurrent validity, discriminative validity and test-retest procedure (Tables 3 and 4).^9^^,^^10^^,^^11^^,^^12^^,^^13^^,^^14^^,^^15^^,^^16^^,^^17^^,^^18^^,^^19^^,^^20^^,^^21^^,^^22^^,^^23^^,^^24^^,^^25^

In order to facilitate the interpretation of the results, the instruments were evaluated together and the characteristics were assessed separately, identifying advantages, disadvantages and psychometric properties.

**Table 3.** Characteristics of articles on spirituality/religiosity (S/R) instruments available for Portuguese language (by year of publication)

Name of scale	Authors*	Journal	Sample type	N
Francis Scale of Attitude Towards Christianity (Portugal)	Ferreira and Neto^9^	Psychol Rep.	University students	323
Strayhorn, Weidman and Larson Religious Scale (Brazil)	Gonçalves^11^	PhD thesis	Breast cancer population	130
Moschella Religious Scale (Brazil)	Gonçalves^11^	PhD thesis	Breast cancer population	130
Private and Social Religious Practice Scale (Brazil)	Drucker^15^	Master Degree dissertation	Depressive older patients	44
Intrinsic Religious Motivation Scale (Brazil)	Drucker^15^	Master Degree dissertation	Depressive older patients	44
Spiritual/Religious Coping Scale (Brazil)	Panzini and Bandeira^20^	Psicol Estud.	Mixed sample	616
Pinto and Pais-Ribeiro’s Spirituality Scale (Portugal)	Pinto and Pais-Ribeiro^22^	Arq Med.	Oncologic patients	426
Brief Santa Clara Strength of Religious Faith (Portugal)	Amado^25^	PhD thesis	Elderly outpatients	194
FACIT-Sp 12 (Brazil)	Guedes^12^	PhD thesis	Diabetes outpatients	54
Spiritual Well-Being Scale (Brazil)	Marques et al.^13^	Aval Psicol.	University students	506
Spirituality Self Rating Scale (Brazil)	Gonçalves and Pillon^14^	Rev Psiq Clín (São Paulo)	Male substance/drug users	138
Aquino’s Religiousness/Spirituality Attitude Scale (Brazil)	Aquino et al.^16^	Psicol Cienc Prof.	High educated population	299
Duke Religion Index (Brazil)	Lucchetti et al.^17^	J Relig Health.	Low income population	383
Pinto and Pais-Ribeiro’s spirituality scale (Brazil)	Chaves et al.^23^	Rev Enferm UFPE Online.	Hemodialysis patients	52
WHOQOL-SRPB (Brazil)	Panzini et al.^19^	Rev Saúde Pública.	Hospital staff and patients	404
Self-reported Religiosity (Brazil)	Lucchetti et al.,^18^	J Rehabil Med.	Rehabilitation patients	110
WHOQOL-100 - SRPB questions (Brazil)	Panzini et al.^19^	Rev Saúde Pública.	Hospital staff and patients	404
Brief Spiritual/Religious Coping Scale (Brazil)	Panzini et al.^19^	Rev Saúde Pública.	Hospital staff and patients	404
Inspirit-R (Brazil)	Veronez et al.^21^	Arq Neuropsiquiatr.	Epilepsy outpatients	50
Daily Spiritual Experience Scale (Brazil)	Oliveira^24^	PhD thesis	Clinical/surgical inpatients	179
Intrinsic Religiousness Inventory (Brazil)	Taunay et al.^10^	Rev Bras Psiquiatr.	(a)Psychiatric and (b)University students	(a) 102 (b) 323

*Only authors who validated their instruments in Portuguese are listed in the table. For original articles and authors, see Discussion.

**Table 4.** Psychometric properties of spirituality/religiosity (S/R) instruments available for Portuguese language

Name of scale	Number of items	Translation process	Cross-cultural adaptation	Internal consistency	Concurrent validity	Discriminative validity	Test-retest procedure
Francis Scale of Attitude Towards Christianity(P)	24	Yes	Yes	0.96	No	No	No
Strayhorn, Weidman and Larson Religious Scale (B)	9	Yes	Yes	No	No	No	No
Moschella Religious Scale (B)	34	Yes	Yes	No	No	No	No
Private and Social Religious Practice Scale (B)	10	Yes	Yes	N/A	No	No	No
Intrinsic Religious Motivation Scale (B)	12	Yes	Yes	No	No	No	No
Spiritual/Religious Coping Scale (B)	87	Yes	Yes	0.97	Yes	Yes	No
Pinto and Pais-Ribeiro’s Spirituality Scale (P)	5	N/A	N/A	0.74	Yes	Yes	No
Brief Santa Clara Strength of Religious Faith (P)	5	Yes	Yes	0.93	Yes	No	No
FACIT-Sp 12 (B)	12	Yes	Yes	No	No	No	No
Spiritual Well-Being Scale (B)	20	Yes	Yes	0.92	No	No	No
Spirituality Self-Rating Scale (B)	6	Yes	Yes	0.83	No	Yes	No
Aquino’s Religiousness/Spirituality Attitude (B)	15	N/A	N/A	0.87	Yes	Yes	No
Duke Religion Index (B)*	5	Yes	Yes	0.75	Yes*	Yes	Yes*
Pinto and Pais-Ribeiro’s Spirituality Scale (B)	5	N/A	Yes	0.64	No	No	No
WHOQOL-SRPB (B)	32	Yes	Yes	0.96	Yes	Yes	Yes
Self-reported Religiosity (B)	1	Yes	Yes	N/A	No	No	No
WHOQOL-100 - SRPB questions (B)	4	Yes	Yes	0.84	Yes	Yes	Yes
Brief Spiritual/Religious Coping Scale (B)	49	Yes	Yes	0.93	Yes	Yes	No
Inspirit-R (B)	7	Yes	Yes	No	No	No	No
Daily Spiritual Experience Scale (B)	16	Yes	Yes	0.91	No	No	No
Intrinsic Religiousness Inventory (B)	10	N/A	N/A	0.96	Yes	Yes	Yes

N/A = not applicable; P = validated for Portugal; B = validated for Brazil. *Recently, a study showed good concurrent analysis and test-retest reliability relating to the Duke Religion Index was published. Since this study was accepted for publication after November 2011, it was not included in our systematic searches but we decided to report its findings here.^42^

### Evaluation of general instruments

In this review, 20 instruments available for measuring S/R in the Portuguese language were assessed. Most of these tools (90%) had previously been developed (n = 3), translated and/or validated for Brazilian culture and published in scientific journals (60%) or as MSc/PhD theses (25%) within Brazilian universities. The majority of the articles providing validation in Portuguese were published in 2011, while the first was published in 2002 (Portugal). This finding reveals that the field of S/R research is new in Portuguese-speaking countries and has been increasing over the last decade. Forty-five percent of the instruments evaluate religiosity (organizational, non-organizational and/or intrinsic), 40% evaluate spirituality, 10% evaluate religious/spiritual coping and 5% evaluate both spirituality and religiosity.

The subjects evaluated by these instruments were drawn from a wide range of settings and included university students, inpatients and oncological patients. Nevertheless, other subjects such as epileptic, diabetic, breast cancer and rehabilitation patients were also included in these studies. The mean number of participants recruited for each study was 241.5 (standard deviation, SD = 36.7), with sample sizes ranging from 44 to 616.

Interestingly, 3 out of the 20 instruments (15%) were originally created in Portuguese, which denotes that there is a need for specific scales that take into account the religious background of Portuguese-speaking countries, which tends to differ from that of other countries. All the other 17 instruments had been translated and adapted to Portuguese, and 12 out of these 17 (70.5%) presented confirmed internal consistency.

However, the majority of the studies had not been subjected to an associated in-depth psychometric analysis. Only 40% of the instruments presented confirmed concurrent validity and 45% discriminative validity, while 15% had test-retest procedures available.

### EVALUATION OF SPECIFIC INSTRUMENTS

The instruments below are listed according to year of publication of their Portuguese versions. References for the original authors of each instrument are also included after citing the Portuguese version:

1. Francis Scale of Attitude Towards Christianity: ^9^^,^^10^^,^^11^^,^^12^^,^^13^^,^^14^^,^^15^^,^^16^^,^^17^^,^^18^^,^^19^^,^^20^^,^^21^^,^^22^^,^^23^^,^^24^^,^^25^^,^^26^ a 24-item Likert-type instrument concerned with affective responses toward God, Jesus, the bible, prayer and church. Each item is assessed on a five-point scale from “agree strongly” to “disagree strongly”. Church attendance was assessed on a five-point scale from “never” to “nearly every week” and personal praying was assessed on a five-point scale from “never” to “daily”. Advantages: complex measurement of affective response towards Christianity. Disadvantages: not suitable for other religious backgrounds (Christianity-focused); it is extensive but does not have a test-retest procedure or adaptation to Brazilian culture.
2. Strayhorn Religious Scale: ^11^^,^^12^^,^^13^^,^^14^^,^^15^^,^^16^^,^^17^^,^^18^^,^^19^^,^^20^^,^^21^^,^^22^^,^^23^^,^^24^^,^^25^^,^^26^^,^^27^ a nine-item religiousness scale without reference to exclusive denominational practices. It evaluates common church practices (attendance, monetary giving and service) and assesses beliefs and practices relating to having a personal relationship with God (awareness of a religious purpose).^28^ Advantages: simple, fast and easy to apply. Disadvantages: the Portuguese version has no test-retest procedure and has not been validated for internal consistency or concurrent and discriminative ability. The scale evaluates religiosity as opposed to spirituality, and does not separate organizational, non-organizational and intrinsic religiosity. The original scale has 12 questions, which is different from the Portuguese translated version with 9 questions. During the cross-cultural adaptation, the authors created a modified version associating the Strayhorn, Weidman and Larson Religious scale with the Moschella scale, containing 25 items, which they named the Gonçalves, Ferraz and Giglio scale.^11^
3. Moschella Religious Scale:^11^^,^^29^ a 34-item scale that evaluates religious involvement (self-reported religiosity and religious attendance), religious struggle and religious coping, among others. Advantages: this is a broad instrument covering some important issues such as religious coping and religious struggle. Disadvantages: the scale was tailored for use among cancer patients and has some items that are related to diseased individuals. The Portuguese version has no test-retest procedure and has not been validated for internal consistency or concurrent and discriminative ability. The scale evaluates religiosity, but not spirituality, and does not separate organizational, non-organizational and intrinsic religiosity. There are some questions such as “Do you believe in elves, fairies and wizards?” or “Have you ever sought help from some psychic or fortune teller?” that seem to be out of context. As already stated, during the cross-cultural adaptation, the authors created a modified version associating the Strayhorn, Weidman and Larson Religious scale with the Moschella scale, containing 25 items, which they named the Gonçalves, Ferraz and Giglio scale.^11^
4. Private and Social Religious Practice Scale:^15^^,^^30^^,^^31^ a 10-item instrument that assesses the frequency of prayer, religious attendance (i.e. attending a church or temple, and religious meetings), reading religious literature, watching religious programs on television, religiosity in the last decade and friends in religion, among others.^18^ Advantages: simple, fast and easy to apply, and has several qualitative items such as: “Why do you pray?” and “Why has your religiosity increased in the last 10 years?” Disadvantages: does not measure intrinsic religiosity, but only “private and social religious practice”; there is no associated published paper validating it; and some items are confusing (religious attendance: every day, once a week, once in a while, never; in this case, if the person attends twice a week, none of the options fit).
5. Intrinsic Religious Motivation Scale (adapted):^15^^,^^32^ a 12-item scale in a Likert-like format evaluating two different aspects of religiosity: intrinsic and extrinsic. The score ranges from 12 to 60, and higher scores indicate more extrinsic religiosity. Advantages: brief and seeks to separate intrinsic and extrinsic religiosity using the same questionnaire. Disadvantages: no published papers validating the scale are available; and it only separates extrinsic-intrinsic religiosity and does not measure how religious the person is. The scale is complex for less-educated individuals. The Portuguese version was adapted from the original by adding two extra questions (original psychometric qualities should not be used in this version).
6. Spiritual/Religious Coping Scale (SRCOPE):^20^^,^^33^ an 87-item with subscales that are intended to provide researchers with a tool for measuring the myriad manifestations of religious coping and to help practitioners better integrate religious and spiritual dimensions into treatments.^33^ Each of the subscales consists of items to which participants respond on a five-point Likert scale ranging from one, “not at all”, to five, “a great deal”. Advantages: it provides a complex and detailed analysis of spiritual and religious coping (including negative coping) with good psychometric qualities. Disadvantages: a very extensive instrument that is difficult to use in epidemiological studies and low-income populations and is time-consuming.
7. Pinto and Pais-Ribeiro’s spirituality scale:^22^^,^^23^ an instrument consisting of five items centered on two dimensions: one associated with belief and the other associated with hope/optimism. The responses are of Likert type, given on a scale of four alternatives, from “do not agree” to “strongly agree”.^34^ Advantages: simple and easy-to-use instrument, quickly applied, created originally in Portuguese. Disadvantages: three out of the five facets included in the instrument, i.e. hope, change in life and value, have been associated with religious involvement, but are not themselves religious/spiritual facets. The Brazilian version^23^ has neither a test-retest procedure nor concurrent and discriminative validation.
8. Brief Santa Clara Strength of Religious Faith:^25^^,^^35^ a five-item questionnaire designed to measure strength of religious faith on a four-point scale, without taking the respondent’s religious background into consideration.^35^ Advantages: easy to administer and score and straightforward to follow; widely used worldwide. Disadvantages: The Portuguese version lacks a test-retest procedure and discriminative validation; there is no version adapted to Brazilian culture.
9. FACIT-Sp 12:^12^^,^^36^ this consists of 12 items and three sub-domains of spiritual wellbeing, thus facilitating in-depth exploration of the components constituting spiritual well-being (peace, meaning and faith).^37^ All of the FACIT-Sp questionnaires were designed for self-administration and use a five-point Likert-type scale to measure patient-reported HRQOL (0 = not at all, to 4 = very much). Advantages: has been used in numerous published papers worldwide. Disadvantages: the Portuguese version lacks a test-retest procedure, internal consistency analysis and concurrent and discriminative validation. Some of the facets included in the instrument, e.g. “I have a reason for living”, “I feel peaceful” and “My life has been productive” have been associated with religious involvement, but do not in themselves denote religiousness/spirituality.
10. Spiritual Well-Being Scale:^13^^,^^38^ a 20-item self-administered scale designed to measure spiritual wellbeing in both its religious (RWB) and existential (EWB) senses. Two subscales are included: (I) RWB, 10 religious items contain a reference to God; (II) EWB, 10 items with no reference to God. In order to control for response-set problems, half of the items from each subscale were worded with positive meanings and half with negative meanings. In Brazil, the scale was translated and adapted for use among university students and yielded an internal consistency of 0.92. No test-retest, concurrent or discriminative validity analyses have been performed. Advantages: much research has used this scale worldwide and it is a brief instrument. Disadvantages: each question on the RWB subscale includes the word “God,” although reviews claim that it is nonsectarian. The Portuguese version has no test-retest procedure, or concurrent and discriminative validations.
11. Spirituality Self-Rating Scale:^14^^,^^39^ this reflects individuals’ orientation towards spirituality, i.e. whether they consider questions concerning the spiritual/religious dimension to be important, and how they apply this in their lives. The scale consists of six statements, which have Likert-type responses ranging from totally agree to totally disagree.^34^ Advantages: simple, fast and easy to apply. The instrument does not include secularism or quality-of-life measurements, but only spiritual issues. Disadvantages: the Portuguese version currently has no test-retest or concurrent validity.
12. Aquino’s Religiousness/Spirituality Attitude Scale:^16^^,^^40^ a 15-item instrument that evaluates religious attitude (“I feel attached to a higher being”, “I attend the celebrations of my religion/spirituality” and “I seek to know the doctrines or religious precepts”). The participants respond using a five-point Likert scale ranging from 1 = never to 5 = always. Advantages: created originally in Portuguese; complex yet not extensive. Disadvantages: does not have any test-retest procedure available; evaluates religiosity more than spirituality; and does not separate organizational, non-organizational and intrinsic religiousness.
13. Duke Religion Index:^17^^,^^41^ a five-item measure of religious involvement, which yields three subscales: (I) organizational religious behavior (one item); (II) non-organizational religious behavior (one item); and (III) intrinsic religious motivation (three items). The response options are on a five or six-point Likert scale. Advantages: Simple and easy (validated in a low-income population), fast to apply, covers three religious dimensions, is widely used worldwide and has good psychometric qualities. Recently, a study^42^ showed good concurrent analysis and test-retest reliability (Intraclass Correlation Coefficient > 0.90) relating to this index. Since this study was accepted for publication after November 2011, it was not included in our systematic searches but we decided to report its findings here. Disadvantages: does not evaluate spirituality.
14. WHOQOL-SRPB:^19^^,^^43^ this contains 32 questions covering eight facets of spirituality, religion and personal beliefs relating to health and quality of life, with final scores ranging from four to 20. Advantages: extensively studied and validated in Brazil with good psychometric qualities; widely used worldwide. Disadvantages: complex and extensive; some of the facets included in the instrument, e.g. meaning of life, awe, wholeness & integration and hope & optimism, have been associated with religious involvement, but they do not in themselves denote religiosity/spirituality.^44^ Moreover, the instrument was primarily designed to evaluate quality of life and not spiritual beliefs.^45^
15. Self-reported religiosity:^18^ also known as subjective religiosity,^46^ and is assessed by asking respondents to rate the importance of religion to them with possible answers: “very important”, “somewhat important”, “little important” and “not at all important”. Advantages: simple and easy to measure, particularly for less educated individuals and in epidemiological studies. It is devised to measure intrinsic religiosity. Disadvantages: difficult to measure a multiple complex issue such as religiosity. Since it is not a scale, only a translation and cross-cultural adaptation are available.^18^
16. WHOQOL-100 - SRPB questions:^19^^,^^47^ the WHOQOL-100 instrument contains four questions (domain VI) evaluating spirituality, religiousness and personal beliefs. The response options are on a five-point Likert scale from “not at all” to “an extreme amount”. Advantages: brief instrument, easy to complete and offering good psychometric qualities. Disadvantages: some of the facets included in the instrument, e.g. meaning of life and optimism, have been associated with religious involvement, but do not in themselves denote religiosity/spirituality,^44^ and the instrument was primarily designed to evaluate quality of life and not spiritual beliefs.^45^ It is less complex than the WHOQOL-SRPB.
17. Brief Spiritual/Religious Coping Scale:^19^^,^^48^ a reduced form of the SRCOPE. This scale includes 49 items divided into two dimensions (positive SRCOPE, 34 items, seven factors; and negative SRCOPE, 15 items, four factors), four general indices and 11 factorials from the means of the items, with results from 1 to 5 for SRCOPE use. Advantages: shorter but complex and detailed analysis of spiritual and religious coping with good psychometric qualities. Disadvantages: remains a very extensive instrument that is difficult to use in epidemiological studies and low-income population; time-consuming. A good alternative to SRCOPE.
18. Inspirit-R^21^^,^^49^ - The Index of Core Spiritual Experience: this questionnaire contains seven items and is designed to identify more intense and concrete experiences relating to the existence of God or a Higher Power, among respondents. The seventh item consists of a list of 12 types of religious experiences, and patients are asked whether they have had any of these experiences, thereby convincing them that God exists. For each item of the questionnaire, which all carry the same weight, the patient gives a rating from 1 to 4. Advantages: helps quantify some perceived aspects of spirituality and is a brief instrument. Disadvantages: the Portuguese version lacks a test-retest procedure, internal consistency analysis and concurrent/discriminative validations.
19. Daily Spiritual Experience Scale (DSES):^24^^,^^50^ a 16-item self-reporting measurement designed to assess ordinary experiences that might have a connection with the transcendent in daily life. It includes constructs such as awe, gratitude, mercy, sense of connection with the transcendent and compassionate love. The scale also includes measurements of awareness of discernment/inspiration and a sense of deep inner peace.^51^ Advantages: the DSES is better accepted by non-religious researchers and respondents than many scales, partly due to the substantial section of non-explicitly religious questions.^51^ The scale is a brief and quick-to-apply measure that is widely used worldwide. Disadvantages: some of the facets included in the instrument, e.g. “I feel deeper inner peace or harmony” and “I feel a selfless caring for others”, have been associated with religious/spiritual involvement, but they do not in themselves denote religiousness/spirituality. The Portuguese version has no test-retest procedure or concurrent and discriminative validations.
20. Intrinsic Religiosity Inventory:^10^ A 10-item Likert-type scale on which each statement is followed by five possible responses from 1 = never to 5 = always, which evaluates intrinsic religiosity (“People find their master motive in religion. Other needs, strong as they may be, are regarded as of less ultimate significance”).^52^ Advantages: simple and easy instrument that is fast to apply and was created originally in Portuguese. Has good psychometric qualities. Disadvantages: does not evaluate spirituality or organizational and non-organizational religiosity.

## DISCUSSION

Surprisingly, there are 20 S/R instruments for health research in Brazil and Portugal. Before this analysis, a lower number of instruments was expected due to the few Brazilian and Portuguese studies in this area over the last few decades. Analysis in greater detail revealed that most of the instruments were translated or created after 2005, which coincides with the beginning of spirituality courses in Brazilian medical schools,^53^ as well as an increased number of studies on this issue. This dramatic increase in numbers reflects the potential of Portuguese-speaking countries in this field.

According to the present analysis, most instruments in Portuguese had been translated or developed but not fully validated, and offered good psychometric qualities. According to Polit et al.,^54^ “the reliability of an instrument is a property not of the instrument but of the instrument when administered to a certain sample under certain conditions”. Therefore, in the future, all instruments should be validated for a wide variety of different samples and in relation to all psychometric properties, and should have concurrent and discriminative validity as well as a test-retest procedure.

This study has some limitations that should be highlighted. Firstly, only the instruments published in PubMed, Embase, Cochrane Library, Google Scholar, SciELO and Lilacs were evaluated. Although these represent the largest and most appropriate databases for review, other measurements published in journals not covered by these databases may exist. In addition, any other instruments published in books or presented in congresses were not included in the final analysis. Secondly, as pointed out by Monod et al.,^7^ the criteria used to include instruments in this type of review are subject to criticism, since spirituality remains a broad, complex and multidimensional concept that lacks definitional consensus. The exclusion of instruments designed to assess dimensions only loosely related to spirituality seems logical (i.e., hope or peace), but not considering instruments measuring broad concepts such as purpose or meaning in life remains a matter of debate.

## CONCLUSIONS

Currently, 20 instruments for measuring spirituality and/or religiosity are available in the Portuguese language. Most of these instruments have been translated/adapted or developed in Brazil and offer good internal consistency. Nevertheless, few instruments have been fully assessed in relation to psychometrical qualities, or have a test-retest procedure or confirmed concurrent and divergent validity. Further validation studies are needed in order to fully assess these Portuguese-language instruments on a range of different samples.
